# Too Late to Reverse: An Atypical Postpartum Case of Acute Necrotizing Pancreatitis with Refractory ARDS Despite ECMO Support

**DOI:** 10.3390/life15091347

**Published:** 2025-08-26

**Authors:** Mihaly Veres, Sanziana Flamind Oltean, Sorin Pascanu, Mihaela Butiulca, Oana Elena Branea, Alexandra Elena Lazar, Bianca Liana Grigorescu

**Affiliations:** 1 Doctoral School of Medicine and Pharmacy, George Emil Palad University of Medicine, Pharmacy, Science and Technology of Targu Mures, 540142 Targu Mures, Romania; 2 Department of Intensive Care, Emergency Institute for Cardiovascular Diseases and Transplantation, 540136 Targu Mures, Romania; 3 Department of Anesthesiology and Intensive Care, County Emergency Clinical Hospital of Targu Mures, 540136 Targu Mures, Romania; 4 Anesthesiology and Intensive Care Department, George Emil Palade University of Medicine, Pharmacy, Science, and Technology of Targu Mures, 540142 Targu Mures, Romania; mihaela.budrescu@umfst.ro (M.B.); oana.branea@umfst.ro (O.E.B.); alexandra.lazar@umfst.ro (A.E.L.);

**Keywords:** acute pancreatitis, necrotizing pancreatitis, ARDS, ECMO, postpartum

## Abstract

During pregnancy and in the postpartum period, several diseases may arise or become exacerbated. Acute pancreatitis incidence during pregnancy is similar to the general population but increases in the first two years after delivery. This case report describes the evolution of necrotizing acute pancreatitis in a 30-year-old woman five months postpartum, with an atypical debut of acute pancreatitis, where the high levels of triglycerides caused by hormonal changes in the late postpartum period overlapped with an underlying hyperlipemia. Despite aggressive, multidisciplinary care, including surgical necrosectomy, continuous renal replacement therapy (CRRT), protective ventilation, and venovenous extracorporeal membrane oxygenation (VV-ECMO), the prognosis was influenced by the hormonal changes both secondary to hypothalamic–pituitary–adrenal dysregulation and the postpartum hormonal changes, leading to an altered inflammatory response, evolution to MODS, ultimately resulting in death. The case highlights the complex interplay between postpartum immune and hormonal changes and the systemic inflammatory response of pancreatitis, emphasizing the critical need for postpartum-specific guidelines in managing acute pancreatitis, particularly regarding early risk stratification in order to prevent this pathology and its complications.

## 1. Introduction

Postpartum pancreatitis is a rare but potentially life-threatening condition that can lead to severe complications, including ARDS and MODS, due to a pronounced systemic inflammatory response. The postpartum period poses unique challenges for managing pancreatitis because of physiological changes in the maternal body and the potential impact on treatment response.

### 1.1. Incidence and Etiology

The incidence of acute pancreatitis (AP) during pregnancy is reported to be around 1 in 1000 to 5000 pregnancies, similar to the general population, but it increases in the first 2 years after delivery. A recent meta-analysis reports a 3% increase in AP incidence per year in the general population [1,2,3]. The most common causes of AP are gallstones and alcohol use, hypercalcemia, and hypertriglyceridemia, followed by autoimmune, idiopathic, and drug-induced pancreatitis. During pregnancy, cholelithiasis is responsible for more than 65% of cases, followed by hypertriglyceridemia [1,4,5]. Severe gestational hypertriglyceridemia has a maternal mortality rate of approximately 20% [6].

### 1.2. Pathophysiology

The pathophysiological cascade of severe pancreatitis involves the release of pancreatic enzymes and subsequent systemic inflammatory response, leading to multi-organ dysfunction. In the postpartum period, immune and hormonal changes may exacerbate the severity of the inflammatory response [7,8,9].

Among the hormonal changes during pregnancy and the postpartum period, a threefold rise in plasma cortisol levels and hypothalamic–pituitary–adrenal axis insufficiency are common, and they are associated with increases in peripheral inflammatory cytokines leading to immune dysregulation. A physiological rise in cholesterol and triglycerides is seen, especially in the third trimester, but in women with underlying hypertriglyceridemia, they remain elevated 12 months postpartum [10,11,12].

The high levels of progesterone during pregnancy or with hormone replacement therapy impair gallbladder contractility, leading to a delayed emptying and favoring gallstone lithiasis and pancreatitis [13].

Critically ill patients develop a hypothalamic–pituitary–adrenal insufficiency, leading to important neuroendocrine dysfunctions. When acute pancreatitis develops in the postpartum period, hormonal changes secondary to hypothalamic–pituitary–adrenal dysregulation overlapping the peripartum and postpartum hormonal changes lead to significant changes in immune response, which are responsible for an increase in the severity of the disease and poor prognosis.

Development of organ failure is the major cause of mortality in AP, respiratory failure and ARDS being the most common and well-established complications that occur. The last decades brought a new understanding of the pathogenesis, diagnosis, and treatment of AP-related ARDS, but despite improvements in access to care, imaging, and interventional techniques, AP still has a high morbidity and mortality [14].

In ARDS, a higher hormonal response is observed, consisting of pronounced activation of copeptin, reduced levels of IGF1, activation of prolactin, and suppression of the thyroid axis. The level of cortisol rises due to the activation of the hypothalamic–pituitary–adrenal axis and secondary to the suppression of albumin and cortisol-biding proteins. On the short term, these changes are beneficial but lead to the development of a form of central adrenal insufficiency. In the chronic phase of stress response, all neuroendocrine axes are suppressed [15,16,17].

Alterations in the gut barrier lead to increased gut permeability, with bacteria translocation and endotoxin diffusion that potentiates lung injury through the gut–lymph–lung axis. In 2023, Hu et al. conducted the first study that reported that the degree of gut barrier injury is one of the prognostic factors in AP [18,19,20,21,22].

The development of organ failure is a major complication of AP, ARDS, and respiratory failure, increasing mortality to 30–40%. The release of pro-inflammatory factors in the bloodstream, like IL-1, IL-6, TNF-alpha, platelet-activating factor, adhesion molecules, chemokines, and reactive species of oxygen, leads to epithelial and endothelial injury, the disruption of the alveolar–capillary barrier, and increased capillary permeability, causing an initial exudative phase resulting in diffuse alveolar damage, followed by a fibro-proliferative phase resulting in fibrosis [23,24].

The ventilation–perfusion miss-match is the leading cause for an increase in peripheral oxygen demand and tissue hypoxemia. Secondary to anaerobic metabolism, ATP production decreases and mitochondrial dysfunction appears, leading to multiple organ failure.

V-V ECMO is recommended in refractory but reversible respiratory failure unresponsive to conventional treatment. In a patient with low oxygen saturation (SaO_2_ < 90% with FiO_2_ at 100%) and elevated carbon dioxide levels (PaCO_2_ > 45 cm H_2_O, with tidal volume 200 mL/kg/min), with a PaO_2_/FIO_2_ ratio of 70–80 mmHg, Murray score > 3, and pH < 7.2, VV-ECMO should be considered [25,26,27].

### 1.3. Particularity of the Case

This case represents a rare and severe progression of postpartum necrotizing pancreatitis complicated by refractory ARDS and multiorgan dysfunction, despite aggressive and multidisciplinary management. The patient underwent VV-ECMO, CRRT with Oxiris and Cytosorb hemoadsorption, and repeated bronchoscopy for clots and atelectasis, showcasing the full spectrum of advanced critical care interventions. The complexity of this case lies in its intersection of postpartum physiology, systemic inflammatory response, and respiratory failure, and underscores the limitations of the most advanced life-support therapies when faced with overwhelming systemic inflammation. It also highlights the need for more research and clear protocols in managing similar critical postpartum cases.

The unique and particularly striking aspect is the development of severe necrotizing pancreatitis five months postpartum, progressing to refractory ARDS despite full-spectrum critical care, including ECMO, a scenario not commonly reported in the literature.

The case highlights the challenges of managing severe postpartum pancreatitis and the limitations of advanced supportive therapies in reversing MODS. A review of the literature emphasizes the necessity of tailored management protocols and further research into effective therapies for ARDS and inflammatory complications during the postpartum period.

## 2. Case Presentation

### 2.1. Patient History, Onset of the Disease and Clinical Findings

Our case presents an acute necrotizing pancreatitis with a poor prognosis occurred in a 30-year-old woman during the late postpartum period. Our patient was five months postpartum after a full-term vaginal delivery. She had an uncomplicated pregnancy and no significant medical history, except for being overweight (weight: 73 kg, height: 1.68 m, and a BMI of 25.86) and having higher levels of triglycerides (150–200 mg/dL). The pregnancy was regularly monitored by the obstetrics-gynecology specialist. During pregnancy, the patient had elevated triglyceride levels (180–250 mg/dL). The patient had no history of alcohol use or chronic medication.

The patient presented to the emergency department with severe epigastric pain radiating to the back, nausea, and vomiting. Her laboratory workup revealed elevated serum amylase and lipase levels (amylase: 946 U/L, lipase: 729 U/L). Seric calcium was 3.7 mg/dL. The RUQ US revealed a thickened gallbladder wall without gallstones and no bile duct dilatation, an inhomogeneous, enlarged, and unclearly demarcated pancreas, and peripancreatic fluid. The CT scan at admission revealed an enlarged, inhomogeneous pancreas, with hypodense areas diffusely located, associated with marked peripancreatic fuses. No intra- or extrahepatic bile duct dilatation, confirming acute pancreatitis. Additional lab findings indicated elevated inflammatory markers (C-reactive protein (CRP): 394 mg/L, leukocytes: 12.61 10^3^ µ/L). Triglyceride levels were elevated—321 mg/dL.

### 2.2. Admission

The patient was admitted to the Gastroenterology Clinic for conservative treatment according to the international protocols. The AJG (American Journal of Gastroenterology) guidelines recommend moderate to aggressive fluid resuscitation. We opted for moderate fluid resuscitation for the first 48 h, using lactate Ringer 1.5 mL/kg/h, monitoring the MAP, urinary output, and electrolyte levels. Insulin infusion was also administrated, with adjuvant 5% glucose infusion required when blood glucose level fell below 200 mg/dL. Due to the concern of rebound hypertriglyceridemia and the risk of evolving to acute hemorrhagic pancreatitis, heparin was avoided.

At admission, the patient had a CTSI of 4 and a Ranson score of 4 points. At 48 h, the Ranson score was 8 points. The evolution of pancreatitis was monitored by daily laboratory workups and repeated abdominal CT scans.

### 2.3. Evolution

The patient’s evolution, initially favorable, was complicated by a mild respiratory distress episode, requiring ICU admission and treatment, without necessitating mechanical ventilation. After remission, the patient was transferred to the gastroenterology clinic for further medical care.

After 36 days of monitoring and treatment in the Gastroenterology Clinic, the patient’s status deteriorated, developing signs of respiratory failure. The abdominal CT scan revealed multiple organized, encapsulated fluid collections and areas of necrotic changes, with a CTSI of 8, confirming the diagnosis of necrotizing pancreatitis; therefore, the patient underwent emergency laparotomy with pancreatic and retroperitoneal necrectomy. Intraoperative culture results from the peripancreatic collections were sterile. Postoperatively, the patient was admitted to the ICU (Intensive Care Unit).

### 2.4. ICU Admission and Management

**Figure 1:**
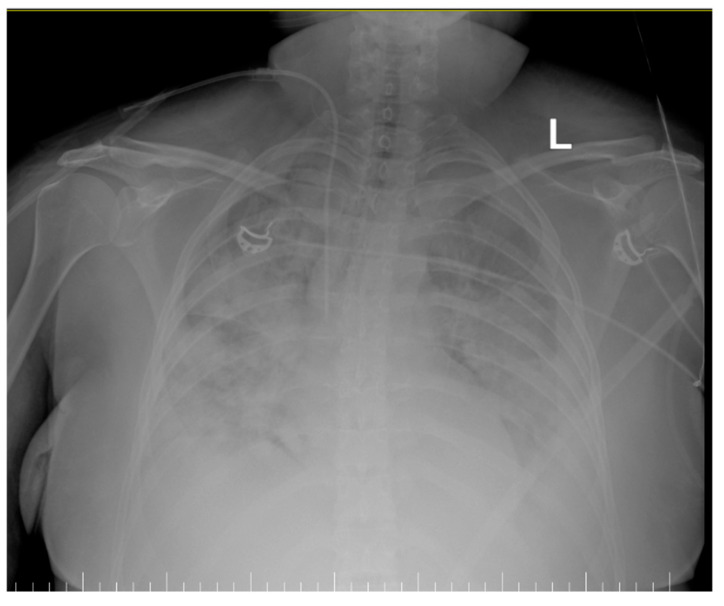
Thoracic X-Ray showing diffused bilateral infiltrates (L-left).

Upon ICU admission, the patient was hemodynamically stable but rapidly developed signs of respiratory distress, requiring supplemental oxygen. Over the next 24 h, her condition worsened, necessitating mechanical ventilation due to hypoxemic respiratory failure consistent with ARDS (PaO_2_/FiO_2_ ratio: 145 mmHg). Chest imaging showed inhomogeneous veiling of both lung fields, diffused bilateral infiltrates, associated with pleural effusion (Figure 1). The Sequential Organ Failure Assessment (SOFA) score was 11, and the APACHE II score was 27.

### 2.5. Advanced Supportive Therapy

Continuous renal replacement therapy (CRRT) with Oxiris hemofiltration was started on day 1 of ICU admission due to acute kidney injury (AKI) with oliguria and rising creatinine levels.

**Figure 2:**
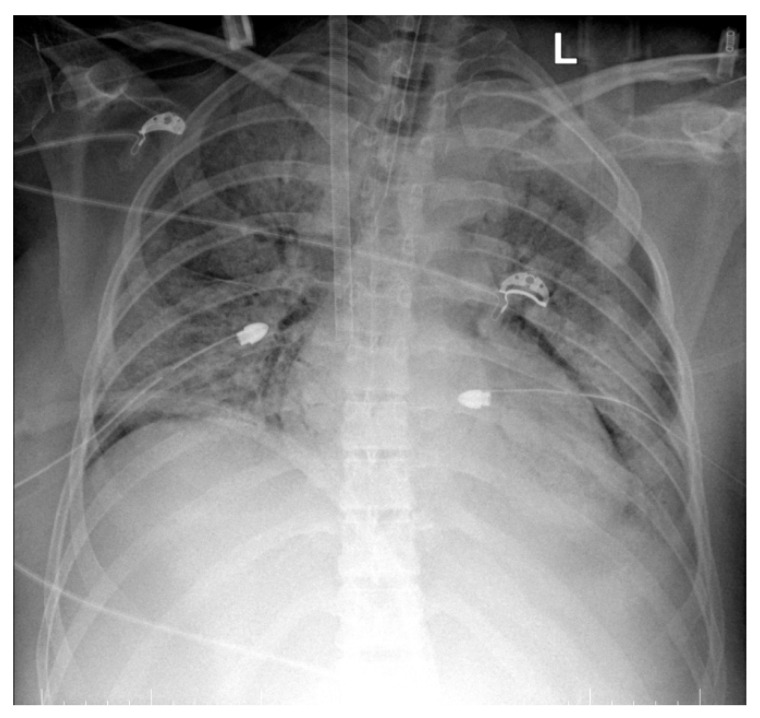
Thoracic X-Rays showing an improvement in the radiological aspect of the lungs after ECMO support was instituted (L-left).

Due to refractory hypoxemia despite optimal ventilator settings (PEEP: 10 cm H_2_O, FiO_2_: 80%), on the 4th postoperative day, venovenous ECMO support was initiated using a femoral–jugular approach. ECMO support was initiated with a flow of 3.2 L, 5000 RPM, and an FiO_2_ of 70%, and the parameters were adjusted based on the patient’s requirements and evolution, with a constant increase in flow and FiO2. After ECMO was instituted, an improvement in ventilation was noticed—PaO_2_ from 40 mmHg to 70–80 mmHg, PaCO_2_ from 85 mmHg to 40–45 mmHg, SpO_2_ from 70 to 75% to 95 to 97%, PaO_2_/FiO_2_ from 40 mmHg to 120–145 mmHg, and a reduction in bilateral infiltrates was revealed by the following thoracic X-rays. (Figure 2.) Changes in respiratory parameters can be seen in Table S1 in the Supplementary Materials.

- Inhomogeneous veiling of the entire left lung field and, respectively, ½ of the right lung field—foci of pulmonary condensation and atelectasis. Bilateral pleural effusion. ECMO cannula at the level of the right internal jugular vein.
- Quasi-complete resorption of pulmonary condensation foci and, respectively, reduction in pleural effusion bilaterally. Lungs with increased transparency. ECMO cannula at the level of the right internal jugular vein.

Cytosorb hemoadsorption therapy was integrated into the ECMO circuit to help reduce circulating cytokine levels, aiming to control the overwhelming systemic inflammatory response. The patient underwent a total of 3 sessions of hemoadsorption.

Repeated CT scans revealed atelectasis of both lungs. Bronchoscopy was performed and revealed an important obstruction of the main bronchus by organized and adherent clots, secondary to the coagulopathy.

Bronchoscopy was performed on the 4th, 6th, 8th, 10th, and 14th days postoperatively, revealing copious secretions and clots. Lavage samples were sent for culture to rule out secondary infection, which returned negative.

Daily laboratory evaluations included complete blood counts, coagulation profiles, renal and liver function tests, arterial blood gases, and inflammatory markers (CRP, procalcitonin, and presepsin). Trends in inflammatory markers (e.g., CRP peaked at 371 mg/L, presepsin at 1852 pg/mL) correlated with clinical deterioration. Carboxyhemoglobin was also monitored, an increase in its values being a sign of impaired liver function, along with hyperlactatemia and high bilirubin levels.

Over the ICU course, the patient developed worsening hepatic dysfunction (bilirubin peaked at 4.95 mg/dL, ALT/AST: 75.2 U/L, 66.3 U/L), worsening coagulopathy (INR: 3.17, platelet count: 81 10^3^ µ/L), and further deterioration in renal function (creatinine peaked at 6.52 mg/dL and urea at 266 mg/dL), requiring escalation of CRRT parameters. The course of laboratory results can be seen in Table S2 in the Supplementary Materials.

During the ICU stay, we did not find any serial positive urine cultures and hemocultures, but due to the elevated trend in leukocytes and inflammatory markers, empiric antibiotic therapy was initiated. To cover both Gram-positive and Gram-negative bacteria, the patient was treated with Meropenem (AstraZeneca UK Limited, Macclesfield, UK) 1 g every 8 h Vancomycin (Fresenius Kabi Romania S.R.L, București, Romania) 20 mg/kgc every 12 h, adjusting the doses according to the creatinine clearance and using maximal dosage while on CRRT. Metronidazole (B. BRAUN MELSUNGEN AG, Melsungen, Germany) 500 mg every 8 h was also administered as empiric therapy to cover a possible anaerobic infection.

On the 13th day after ICU admission, we found a positive bacteriological culture of the endotracheal aspirate for Acinetobacter Baumanii XDR and Klebsiella Pneumoniae XDR, CPE. Colistin (Xellia Pharmaceuticals ApS, Copenhagen, Denmark) 3 MUI every 8 h was initiated, along with antifungal therapy.

The ventilator circuit was changed, along with several changes in the endotracheal tube, central venous catheters, and urinary catheter over the course of the ICU stay. Routine tracheal aspiration was performed to remove secretions.

The antibiogram revealed extended-drug resistance for both isolates for aminoglycosides, extended-spectrum cephalosporins, antipseudomonal carbapenems, fluoroquinolones, penicillins, and b-lactamase inhibitors, sensible only to colistin and cefiderocol.

After receiving the antibiogram, cefiderocol (Shionogi B.V., Amsterdam, The Netherlands) 2 g every 8 h was associated with colistin therapy. Due to the patient’s worsening condition, meropenem and vancomycin were then replaced by teicoplanin (SANOFI-AVENTIS S.p.A., Milan, Italy) at 10–12 mg/kg every 12 h for the first 3 doses, then every 24 h. The antibiotic therapy was guided by an infectious disease specialist. The dosages were adjusted according to the patient’s needs (creatinine clearance, CRRT).

Parenteral nutrition was provided according to the ESPEN guidelines for critically ill patients, and it was started in the early postoperative period in order to prevent villous atrophy and gut bacterial translocation. The NUTRIC score was between 5 and 7 points.

Vasopressor support was needed to maintain adequate mean arterial pressure: norepinephrine (AS KALCEKS, Riga, Latvia) dose: 0.07 µg/kgc/min–1.2 µg/kgc/min; vasopressin (Rizochem Pharmaceuticals, Uttar Pradesh, India) dose: 0.03 UI/min; and association of positive inotropic support was required: dobutamine (Siegfried Hameln GmbH, Hamelin, Germany) (2 µg/kgc/min–20 µg/kgc/min). Continuous sedation was maintained with propofol (B. BRAUN MELSUNGEN AG, Melsungen, Germany) and fentanyl (Akciju sabiedrība Kalceks, Riga, Latvia) infusions to optimize mechanical ventilation and reduce metabolic demand. Propofol doses were adjusted from 5 to 10 mcg/kg/min, fentanyl doses were adjusted from 0.01 to 0.02 mcg/kg/min. Hemodynamics of the patients can be seen in Table S3 in the Supplementary Materials.

Despite ECMO support, oxygenation remained challenging, and evidence of myocardial depression was noted (ejection fraction decreased to 40%).

Considering the high rates of hemorrhagic, renal, cardiovascular, and ECMO circuit-related complications, along with the insufficient data from the literature to support an improvement in the outcome of the patient, conversion from V-V ECMO to V-A ECMO in this case was considered without significant benefits.

Despite maximal supportive measures, including ECMO support, hemofiltration, and multiple multidisciplinary consultations considering management of the disease, the patient succumbed to MODS on the 21st day postoperatively.

**Figure 3:**
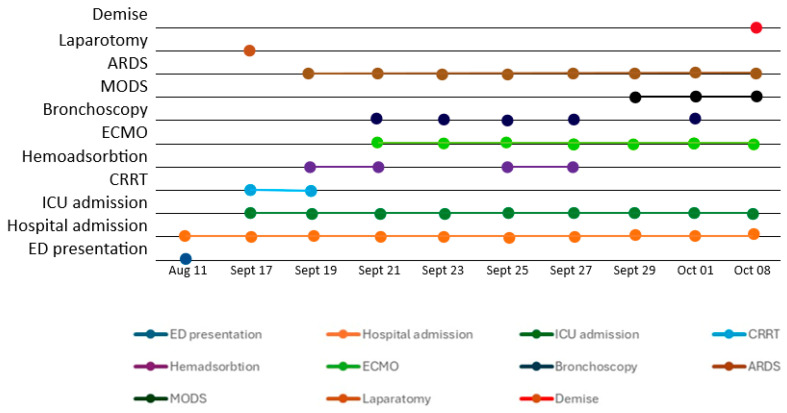
ICU timeline: Postpartum pancreatitis with severe ARDS.

A timeline of the patient’s evolution is presented in Figure 3.

## 3. Discussions

### 3.1. Pathophysiology

As a physiological response to stress, cortisol levels increase throughout pregnancy and continue to increase with advancing labor at term. The increase in cortisol levels is known to have the greatest effect on lipids and lipoproteins, along with an increase in estrogen levels [28,29]. Hyperlipemia is among the first causative factors of AP in the postpartum period, hypertriglyceridemia being caused by hyperestrogen states from pregnancy and the postpartum period [30,31,32].

Our patient presented an atypical debut of acute pancreatitis, where the high levels of triglycerides caused by hormonal changes in the late postpartum period overlapped an underlying hyperlipemia.

The pathophysiological cascade of acute pancreatitis involves the release of pancreatic enzymes, local inflammatory reaction, and subsequent systemic inflammatory response, worsening tissue injury and evolving to SIRS and MODS. The severity of the inflammatory response is exacerbated by the immune and hormonal changes in the postpartum period. The severity of AP predicts mortality, being influenced by the presence of organ failure and necrosis. Mortality reaches two peaks, organ failure being the main cause in the early phase and infection in the late phase [33,34,35,36].

### 3.2. Management

We monitored the trend of the inflammatory response by daily laboratory analysis. Due to the necrotic changes in the pancreas and clinical worsening of the patient, surgical intervention was required. The evolution to ARDS was initially managed conservatively, but due to the severity of respiratory failure, to promote lung rest and recovery, venovenous ECMO support was initiated. The evolution was marked by worsening hepatic dysfunction, renal dysfunction, coagulopathy, and sepsis. Higher carboxyhemoglobin values are common in septic patients as a result of disruption of the heme catabolism caused by liver dysfunction, leading to a reduced lactate clearance, altered inflammatory response, and higher mortality rates [37,38]. By using daily gasometry for monitoring ARDS evolution, we also monitored carboxyhemoglobin levels as an easy-to-calculate and cost-effective bedside biomarker of sepsis in correlation with the patient’s clinical status and severity scores.

The prognosis was influenced by the evolution to MODS, the altered inflammatory response, and the suppression of all neuroendocrine axes common for the critically ill patient.

### 3.3. Implications for Future Practice

A multidisciplinary effort was required with the aim of improving the outcome of the patient, with distinctive roles for intensivists, radiologists, surgeons, gastroenterologists, and obstetricians. Whether earlier initiation of ECMO or more aggressive cytokine modulation would have altered the course remains uncertain. However, the timing of intervention in MODS remains a crucial and controversial area in critical care. There is an urgent need for postpartum-specific protocols for ECMO initiation and inflammatory modulation in the setting of acute pancreatitis.

Understanding the risk factors and pathophysiological mechanisms of postpartum pancreatitis is vital in preventing this complication, along with closer monitoring and better management of patients with an underlying hypertriglyceridemia.

Although there is vast data in the literature considering acute pancreatitis in the early stage of the postpartum period, there is insufficient data considering the late stage of postpartum, which represents the particularity of our case.

### 3.4. Other Cases of Post-Partum Pancreatitis and ARDS: Data from the Literature

We performed a database search on PubMed, using the following keywords: postpartum acute pancreatitis, acute pancreatitis complicated with ARDS, management, and late postpartum acute pancreatitis, with no data range or language limitations. We found several cases of postpartum acute pancreatitis, with different etiologies, none of them concerning the late postpartum period or ARDS, MODS, or ECMO involvement. This aspect underscores the particularity of our case and the complex pathophysiology of a complicated acute pancreatitis progressing to refractory ARDS, intertwining with hormonal and immune changes in the critically ill in the postpartum period, emphasizing the need for further research for this particular postpartum complication.

Maringhini et al. studied the most common etiologies of AP during and after pregnancy, recommending diets for obesity, breastfeeding for at least three months postpartum, and checking triglyceride levels during pregnancy in the prevention of AP [1].

Toth et al. and Kim et al. presented cases of AP developed after C-section, managed with conservative treatment [39,40]. Hofstrand et al. presented a case of a 19-year-old patient diagnosed in the postpartum period with necrotizing acute pancreatitis with gallbladder “sludge” due to microlithiasis, also managed with conservative treatment [20].

Cao et al. [41] reported a case of a 26-year-old patient at 35 weeks of gestation developed hyperlipidemia acute pancreatitis (HLAP) due to high triglyceride levels. The management included aggressive intravenous hydration, bowel rest, antibiotics, insulin, and heparin. Lipid-lowering agents, hemoperfusion, and continuous renal replacement therapy (CRRT) were used to reduce the elevated lipid levels

A single-center retrospective study from Hot et al. [2] has shown a higher incidence of acute biliary pancreatitis related to pregnancy (ABPP), with a relatively high recurrence rate. Supportive treatment, intravenous fluid therapy, probiotics, glutamine, omega-3 fatty acids, and vitamins were used for the initial management, and a multidisciplinary team was required in patients with comorbid factors, HELLP syndrome or preeclampsia

A comprehensive review from Mądro et al. highlights the necessity of a multidisciplinary team, consisting of an obstetrician, gastroenterologist, anesthesiologist, and surgeon, in order to care for both the mother and the fetus. Therapeutic management should be guided by both the severity of pancreatitis and the stage of pregnancy, recommending a strictly conservative management in the first trimester, caution in the administration of painkillers during the pregnancy, and limitations of surgical interventions, the second trimester being the best time to perform laparoscopic cholecystectomy when necessary [42].

Table 1 summarizes data found from published cases of acute pancreatitis during pregnancy and the postpartum period.

**Table 1:** Acute pancreatitis during pregnancy and postpartum period—data from the literature.

Study	Maternal Age	Timing Postpartum	Type of Birth	Etiology	Complications	Interventions	Outcomes
Toth et al. [39]	27-year-old	2 days postpartum	C-section	Idiopathic	-	Conservative treatment	Discharged on the 7th day
Kim et al. [40]	35-year-old	3 days postpartum	C-section	Gallstones	intra-abdominal fluid collections and gastric bleeding	percutaneous drainage, endoscopic hemostasis, angiographic embolization	Discharged on the 31st day
Hofstrand et al. [20]	19-year-old	3 months postpartum	Not mentioned	Gallbladder “sludge”	-	Conservative	Discharged on the 6th day
Cao et al. [41]	26-year-old	35 weeks of pregnancy	Emergency C-section	Hypertriglyceridemia	-	Conservative treatment, insulin infusion, heparin, plasmapheresis and CRRT	Discharged on the 11th day
Shiddapur et al. [43]	25-year-old	30 weeks of pregnancy	Emergency C-section	Pregnancy-induced hypertension complicated with eclampsia	moderate ascites	Conservative treatment and paracentesis	Not mentioned
Lee et al. [44]	38-year-old	1st day after delivery	Vaginal delivery	Hypercalcemia caused by primary hyperparatiroidism	bilateral hydronephrosis caused by renal stones	Conservative treatment, CT-guided percutaneous drainage	Right parathyroidectomy with good recovery at 1 year follow-up
Jallouli et al. [45]	19-year-old	4 weeks postpartum	Vaginal delivery	Idiopathic	-	Conservative management	Discharged on the 14th day
Poo et al. [46]	30-year-old	3 days postpartum	Emergency C-section	insulin-dependent diabetes mellitius from pancreatic islet cell destruction	diabetic ketoacidosis	Conservative treatment + CRRT	long-term insulin requirement
Amir et al. [30]	32-year-old	10 days postpartum	Vaginal delivery	Idiopathic	-	Conservative treatment	Discharged on the 19th day, good recovery at one month follow-up
Dale et al. [47]	31-year-old	32 weeks of pregnancy	C-section	Hypercalcemia caused by primary hyperparatiroidism	-	Conservative management	Discharged on 7th day, referred for parathyroid adenoma resection

AP during pregnancy and the postpartum period is a challenging problem, but ongoing research on acute pancreatitis and acute respiratory injury gives hope of improvement in the management of this severe complication [48].

There is a lack of data in the literature concerning postpartum AP complicated with ARDS. A better understanding of the pathophysiology of ARDS in AP and evidence-based guidelines are needed for the management of AP during and after pregnancy to reduce both maternal and fetal mortality.

### 3.5. Key Learning Points

Postpartum acute pancreatitis is a rare but potentially life-threatening disease, with an unbearable risk of severe complications. The interplay between the hormonal and immunological alterations in the late postpartum period plays a crucial role in the evolution and prognosis of postpartum acute pancreatitis, increasing the risk of poor outcomes due to the rapid progression to organ failure and MODS.

There is no specific therapy available either to treat or to prevent SIRS, organ failure, and MODS. The limitations of advanced supportive therapies, even with ECMO and hemoadsorption, underscore the need for continued research into novel therapeutic approaches to managing the systemic inflammatory response in critically ill patients.

## 4. Conclusions

This case underscores the heightened vulnerability of the early postpartum period to overwhelming inflammatory responses, particularly in the setting of acute pancreatitis. While the rarity of postpartum pancreatitis is notable, the clinical importance lies in how rapidly it progressed to severe ARDS and multiorgan dysfunction syndrome (MODS) despite early detection and a full spectrum of advanced supportive therapies, including VV-ECMO, CRRT, hemoadsorption, and protective ventilation. The patient’s deterioration highlights the limitations of current critical care tools when faced with uncontrolled systemic inflammation. It brings to light the urgent need for earlier risk stratification, inflammatory modulation strategies, and postpartum-specific guidelines for the management of critical illness in this unique patient population. Monitoring of triglyceride levels in pregnant women at risk for hyperlipidemia should be mandatory every trimester, with dietary and lifestyle modifications as measures of prevention for developing acute pancreatitis.

## Data Availability

All the data can be found in the archive of the County Emergency Clinical Hospital of Targu Mures, Romania.

## Supplementary Materials

The following supporting information can be downloaded: https://www.mdpi.com/article/10.3390/life15091347/s1, Table S1: Respiratory Assessment—summarizing the changes in respiratory parameter pre- and post-ECMO, values for respiratory indices and other specialized tests, including COHb, alveolar-arterial gradient [D(A-a)], respiratory index, shunt fraction, Table S2: Laboratory parameters over time—trends in important laboratory values. and Table S3: Hemodynamics of the patient during the course of the ICU stay.

## Abbreviations

ABPP	acute biliary pancreatitis related to pregnancy
AJG	American Journal of Gastroenterology
AKI	acute kidney failure
ALT	alanine aminotransferase
AP	acute pancreatitis
APACHE	acute physiology and chronic health evaluation
ARDS	acute respiratory distress syndrome
AST	aspartate aminotransferase
BP	blood pressure
CRP	C-reactive protein
CRRT	continuous renal replacement therapy
CT	computed tomography
ECMO	extracorporeal membrane oxygenation
FiO_2_	fraction of inspired Oxygen
HELLP syndrome	hemolysis, elevated liver enzymes, and low platelet count
HLAP	hyperlipidemia acute pancreatitis
HR	heart rate
ICU	intensive care unit
IL	interleukin
LDH	lactate dehydrogenase
MAP	mean arterial pressure
MODS	multiorgan dysfunction syndrome
PaO_2_	partial pressure of Oxygen in arterial blood
PaCO_2_	partial pressure of Carbon Dioxide in arterial blood
PCT	procalcitonin
PEEP	positive end expiratory pressure
RR	respiratory rate
RRT	renal replacement therapy
RUQ US	right upper quadrant ultrasound
SaO_2_	oxygen saturation of arterial blood
SIRS	systemic inflammatory response syndrome
SOFA	sequential organ failure assessment
TNF	tumor necrosis factor
VA-ECMO	venoarterial extracorporeal membrane oxygenation
VV-ECMO	venovenous extracorporeal membrane oxygenation
