# Supplementary material for: Too Late to Reverse: An Atypical Postpartum Case of Acute Necrotizing Pancreatitis with Refractory ARDS Despite ECMO Support
Source: Life (Basel). 2025 Aug 26;15(9):1347. doi: 10.3390/life15091347 (PMC12471077; doi:10.3390/life15091347)
Supplement: Supplementary file 1 [file life-15-01347-s001.zip › life-3831582-supplementary.pdf]

Table S1. Respiratory Assessment - summarizing the changes in respiratory parameter pre- and post-ECMO, values for respiratory indices and other specialized tests, including COHb, alveolar-arterial gradient [D(A-a)], respiratory index, shunt fraction.

	**Presentation to the emergency unit**	**Surgical intervention**	**Admission to ICU**	**2nd day ICU**	**4th day ICU – ECMO institution**	**10th day ICU**	**21st day ICU**
Date	11.08	17.09	17.09	19.09	21.09	27.09	08.10
SpO_2 (%)	99	97-99	70-80	70	97	98	70
PaO_2 (mmHg)	116	106	183	40	80	81	50
$PaCO_2$ (mmHg)	27	35	38	85	40	41	41
PaO_2/FiO_2	552	212	105	40	145	86	66
Vent mode	-	PCV	P-SIMV	P-SIMV	P-SIMV	P-A/C	P-A/C
PEEP (cmH_2O)	-	5	5	8	10	10	12
FiO_2 (%)	21	50	60	80	60	80	100
Ventilator RR (/minute)	-	14	14	18	24	22	24
Alveolar-arterial gradient (A-a)	46	197	144.7	393	292	419.4	464
Respiratory Index	0.6	1.1	2.4	4.2	3.9	5	7.4
Shunt Fraction (%)	12.5	18.6	28.6	30	27.1	36.8	47

(SpO2 - Oxygen saturation; PaO2 - partial pressure of Oxygen in arterial blood; PaCO2 - partial pressure of Carbon Dioxide in arterial blood; RR – respiratory rate).

Table S2. Laboratory parameters over time - trends in important laboratory values.

	**Presentation to the emergency unit**	**Surgical intervention**	**Admission to ICU**	**2nd day ICU**	**4th day ICU – ECMO institution**	**10th day ICU**	**21st day ICU**
Date	11.08	17.09	17.09	19.09	21.09	27.09	08.10
Amylase (U/L)	946	384	143	-	27	26	-
Lipase (U/L)	729	289	96	-	36	31	-
Hemoglobin (g/dL)	11.2	9.32	7.5	9.1	8.9	6.5	8.3
CRP (mg/L)	189	213	117	284	371	153	82
Presepsin (pg/mL)	269	-	543	608	671	1852	-
Procalcitonin (ng/mL)	0.3	-	0.5	0.9	11.48	7.4	-

AST (U/L)	54.9	66.3	37	23	16	29	77.2
ALT (U/L)	13.8	75.2	18.9	16	51	17	25.6
Albumin (g/dL)	-	2.13	2.24	2.52	2.6	2.8	3.2
LDH (UI/L)	1998	899	329	403	399	475	729
Creatinine (mg/dL)	1.56	1.78	2.75	1.9	6.52	1.89	1.46
Leukocytes (x10^3/μL)	11.2	23.5	15.5	6.51	11.8	13.2	12.1
Neutrophiles (x10^3/μL)	8.64	21.3	13.71	5.71	10.2	11.9	10.5
Platelets (x10^3/μL)	202	235	234	162	102	83.7	99
Lactate (mmol/L)	0.7	0.8	0.9	0.7	1.1	2.3	16.9
Carboxyhemoglobin (COHb, %)	1.3	1.6	1.7	2.2	1.4	2.9	2.3

(CRP – C-reactive protein; AST – aspartate aminotransferase, ALT – alanine aminotransferase; LDH - Lactate Dehydrogenase).

Table S3. Hemodynamics of the patient during the course of the ICU stay.

	**Presentation to the emergency unit**	**Surgical intervention**	**Admission to ICU**	**2nd day ICU**	**4th day ICU – ECMO institution**	**10th day ICU**	**21st day ICU**
Date	11.08	17.09	17.09	19.09	21.09	27.09	08.10
BP (mmHg)	98/66	60-110/40-80	100-130/55-85	100-115/ 70-85	55-100/ 35-75	110-130/60-70	60/20
MAP (mmHg)	77	63-64	70-80	80-95	75	87-90	33
HR (bpm)	113	110-120	140-160	125-135	130-140	100-120	40-90
Norepinephrine (μg/kgc/min)	-	-	0.07	0.2	0.2	0.1	1.2
Dobutamine (μg/kgc/min)	-	-	-	-	5	10	20
Vasopressin (UI/min)	-	-	-	-	0.03	-	-
Sedation	-	-	x	x	x	x	x

(BP – blood pressure; MAP – mean arterial pressure; HR – heart rate).
